# Data and code for the exploratory data analysis of the electrical energy demand in the time domain in Greece

**DOI:** 10.1016/j.dib.2017.06.033

**Published:** 2017-06-23

**Authors:** Hristos Tyralis, Georgios Karakatsanis, Katerina Tzouka, Nikos Mamassis

**Affiliations:** National Technical University of Athens, School of Civil Engineering, Greece

## Abstract

We present data and code for visualizing the electrical energy data and weather-, climate-related and socioeconomic variables in the time domain in Greece. The electrical energy data include hourly demand, weekly-ahead forecasted values of the demand provided by the Greek Independent Power Transmission Operator and pricing values in Greece. We also present the daily temperature in Athens and the Gross Domestic Product of Greece. The code combines the data to a single report, which includes all visualizations with combinations of all variables in multiple time scales. The data and code were used in Tyralis et al. (2017) [1].

**Specifications Table**

**Table:** 

Subject area	*Energy*
More specific subject area	*Electrical Energy, Energy Forecasting, Electricity Demand*
Type of data	*Table, Figure*
How data was acquired	*Online databases of international and domestic organizations and institutes*
Data format	Raw data in.xls and.hts format.
	Wrangled data in.csv format. Produced after data munging of the.xls files.
	Code in.Rmd format.
	Outcome of code in.html and.docx format
Experimental factors	
Experimental features	
Data source location	*Energy and Gross Domestic Product data refer to Greece. Temperature data refer to Athens*
Data accessibility	*Data is with this article*

**Value of the data**

● Combinations of the data can be used for building an energy-forecasting model.
● Data can be combined with data from other sources to improve the forecasting model.
● The published code and data can be used to reproduce the Tyralis et al. [1] paper.

## 1 Data

We present a collection of electrical energy data and weather-, climate-related and socioeconomic variables in the time domain in Greece. The raw electrical energy data [2] include the hourly energy demand in Greece, the weekly-ahead forecast of the hourly demand and the Ex-ante and Ex-post System hourly Marginal Price (ex-ante and *ex*-post SMPs). The raw weather- and climate-related data include the daily temperature at the Ilioupolis station in Athens, Greece [3]. We also present the Gross Domestic Product of Greece [4]. The reader can find information for the raw data in the “data sources.txt” file (download location, access date etc.), within the “raw data” folder of Supplementary information (see Appendix A).

## 2 Experimental design, materials and methods

We wrangled the raw data and we produced the data in the “data_for_energy_in_Greece” subfolder of the “Electrical energy demand visualization,time domain” folder for further processing. You can find these data in the Supplementary information (see Appendix A), while they are summarized in Table 1.

**Table 1:** Wrangled data included in the Supplementary information of Appendix A.

Variable	Unit	Availability
Demand load	MW	2002/09/01–2016/08/31
Load forecast	MW	2002/09/01–2016/08/31
Ex-ante System Marginal Price (ex-ante SMP)	€/MWh	2002/09/01–2016/08/31
Ex-post System Marginal Price (ex-post SMP)	€/MWh	2002/09/01–2016/08/31
Gross Domestic Product (GDP)	10^6^ €	2002–2015
Gross Domestic Product of hydrological year (GDP_hydr_)	10^6^ €	2002 – 2014 (hydrological years, see Tyralis et al. [1])
Temperature	°C	2005/09/01–2016/08/31

The folder "Electrical energy demand visualization,time domain" includes the code. To run the code:

– Copy the "Electrical energy demand visualization,time domain" folder in your hard disk.
– Open the "Electrical_energy_demand_visualization.Rmd" file using the RStudio.
– Change the in_dir variable to point the location of the folder "Electrical energy demand visualization,time domain"
– Knit the code using the RStudio.
  When knitting the code, using the html outcome option, the outcomes are the following files (depending of the kind of knitting):
– "Electrical_energy_demand_visualization.html"
– "Electrical_energy_demand_visualization.docxl".

Both files include the visualizations presented in Tyralis et al. [1]. After knitting both previous files, they appear in the “code_for_energy_in_Greece” subfolder and then we move them manually to the folder "Code outcome". In the file "Electrical_energy_demand_visualization.html" you can find information about the code e.g. the version of the software and the R packages that were used to produce the visualizations.

## Footnotes

Transparency document Transparency document associated with this article can be found in the online version at http://dx.doi.org/10.1016/j.dib.2017.06.033.
