# Supplementary material for: Data and code for the exploratory data analysis of the electrical energy demand in the time domain in Greece
Source: Data Brief. 2017 Jun 23;13:700–2. doi: 10.1016/j.dib.2017.06.033 (PMC5510486; doi:10.1016/j.dib.2017.06.033)
Supplement: Supplementary file 1 — Supplementary material [file mmc1.docx]

**Declaration of interest**

Conflicts of interest: none.
